# A rare suspected case of chronic nodular granulomatous herpes simplex encephalitis in an adult

**DOI:** 10.1259/bjrcr.20170114

**Published:** 2018-03-14

**Authors:** Lucy Childs, Guan Lim, Andrew Thompson, Timothy R Bates, Lay Kun Kho, Constantine Chris Phatouros

**Affiliations:** 1 Neurological Imaging and Intervention Service Western Australia (NIISwa), Sir Charles Gairdner Hospital, Nedlands, WA, Australia; 2 Stroke Unit, Swan District Hospital, Midland, WA, Australia; 3 School of Medicine and Pharmacology, University of Western Australia, Perth, WA, Australia; 4 Department of Neurology, Royal Perth Hospital, Perth, WA, Australia

## Abstract

Herpes simplex encephalitis is the most common sporadic viral encephalitis in the western world, HSV-1 (herpes simplex virus) being the mostly commonly implicated serotype. The disease is usually monophasic, although patients may relapse weeks, months or years after initial infection. This chronic granulomatous inflammatory process is almost exclusively described in children and rarely forms discrete enhancing parenchymal nodules. We present the clinical and radiological features of an unusual case of chronic nodular granulomatous herpes encephalitis with enhancing “mass-like” nodules in an adult. To the author’s knowledge, this is the first reported case of macroscopic “mass-like” nodular granuloma formation in an adult.

## Introduction

Herpes viruses are associated with a spectrum of neurological diseases with acute, subacute and chronic manifestations. Herpes simplex encephalitis is the most common sporadic viral encephalitis in the Western world and remains one of the most devastating infections of the central nervous system.^1^ Type-1 (HSV-1) and Type-2 (HSV-2) herpes simplex viruses may cause encephalitis with HSV-1 most commonly implicated beyond the neonatal period. The typical disease course is an acute monophonic illness with symptoms such as fever, malaise, headache, delirium and seizures. Uncommonly, patients with herpes simplex encephalitis relapse with recurrent symptoms or signs weeks, months or years after the initial infection.^2, 3^ Chronic disease manifesting as persistent granulomatous inflammation is rarer still particularly in adults.^4–6^ To the author’s knowledge this is the only published case report demonstrating macroscopic nodular granulomatous herpes simplex encephalitis in an adult.

## Clinical Presentation

A 61-year-old female presented to the emergency department with acute onset dysphasia and a 10-day preceding history of fever, vertigo and lethargy. Apart from hypothyroidism, managed with thyroxine, there was no significant past medical or surgical history. The patient was found to have severe expressive and receptive dysphasia. Speech was slow, but fluent, with phonemic paraphrasing errors, with substitution of parts of words or some syllables with other similar-sounding, but nonsensical, sounds and prolific use of jargon. The patient could follow verbal one-step commands but was unable to follow two-stage commands or written commands. She could write, but content was nonsensical. Subtle pronator drift and brisk ipsilateral reflexes were elicited in her right arm in the absence of any sensory or motor weakness. Stroke and encephalitis were both considered in the initial differential diagnosis. Antibacterial and antiviral therapies were initiated.

## Investigations & Imaging Findings

**Figure 1.**
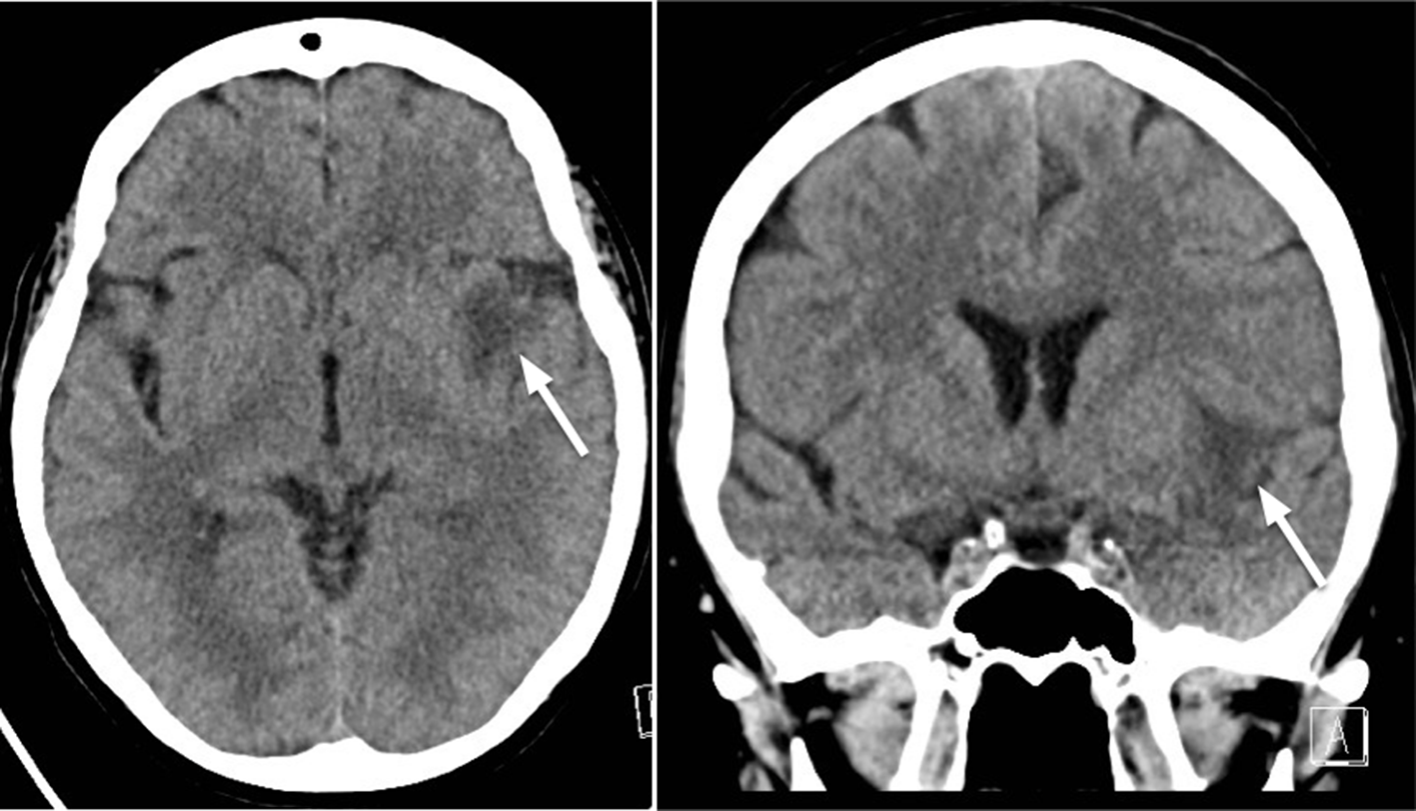
Axial (left) and coronal (right) unenhanced CT of the brain demonstrates hypoattenuation in the anterior left insula ribbon.

CT on the day of admission identified hypoattenuation in the left insula and external capsule without haemorrhage or intravascular thrombus (Figure 1).

 Cerebrospinal fluid (CSF) analysis demonstrated a moderate lymphocytosis with scattered monocytes and lymphocytes. CSF protein was elevated measuring 1.62 g l^−1^. Glucose was within the normal range (3.7 mmol l^−1^) relative to serum glucose and bacterial culture was negative. No malignant cells were seen. Neither cryptococcal antigen nor acid-fast bacilli were identified. Autoantibodies and voltage-gated potassium channel antibodies were within normal range. Viral polymerase chain reaction (PCR) panel was positive for herpes simplex Type-1 DNA, confirming herpes simplex encephalitis. The patient was commenced on i.v. acyclovir.

**Figure 2.**
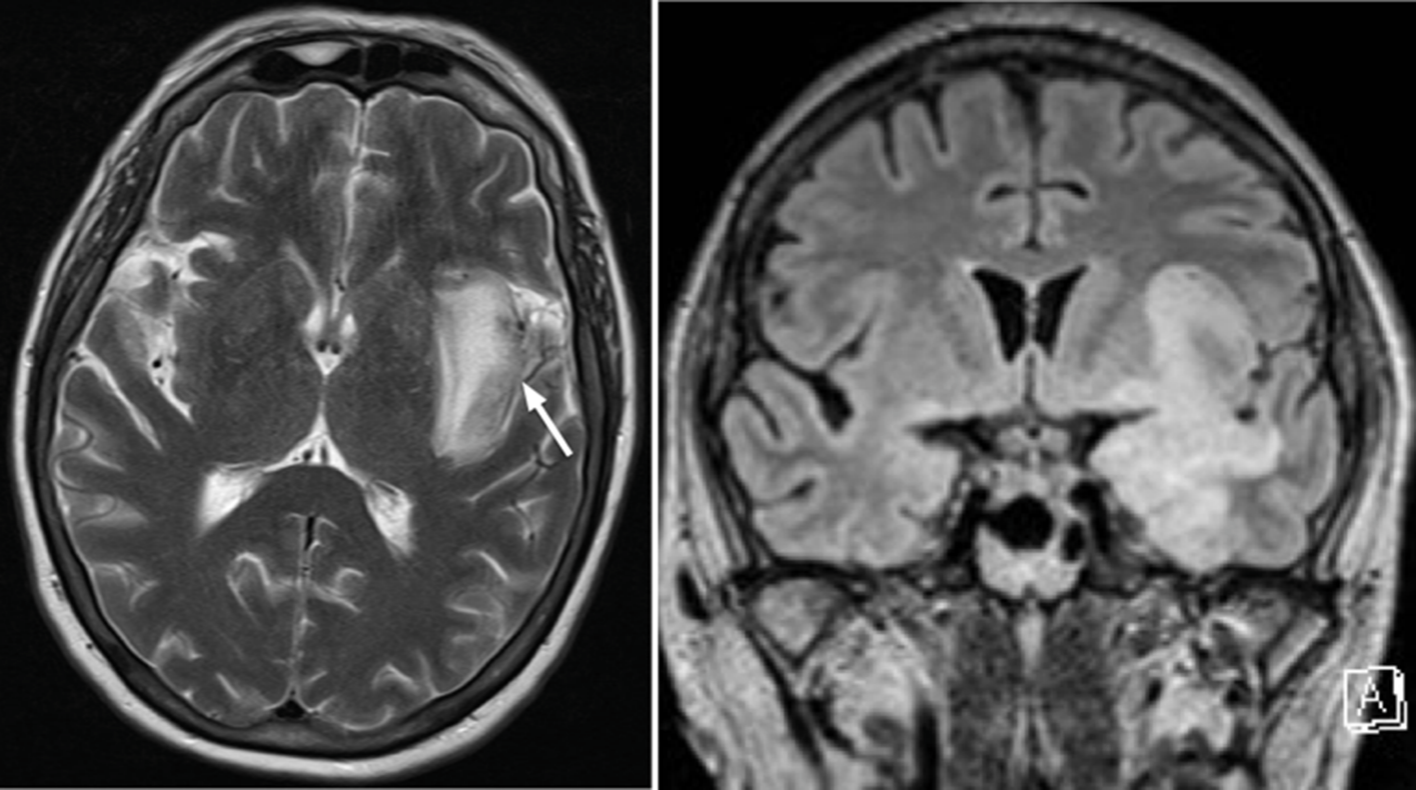
Axial T2 sequence (left) at the time of acute admission identifies T2 hyperintense parenchymal oedema with mild associated mass effect in the left insular region. Corresponding coronal FLAIR (Fluid-attenuated inversion recovery) sequence (right) shows florid signal abnormality in the left mesial temporal lobe, temporal stem, insular and subtle subfrontal signal abnormality.

**Figure 3.**
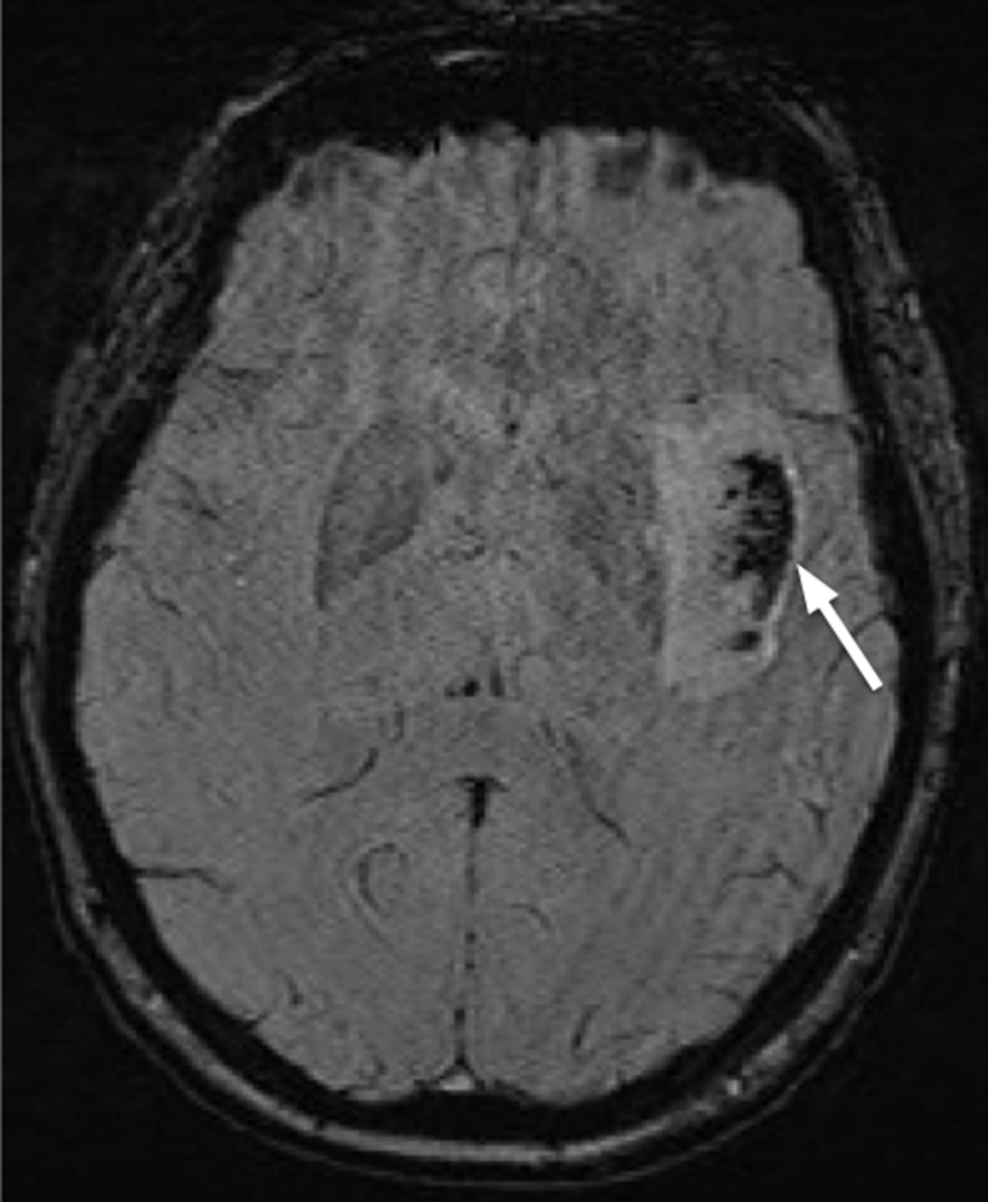
Signal in the left insular region consistent with haemorrhagic change on the susceptibility-weighted image. Haemorrhagic necrosis is a common feature in HSV encephalitis. HSV, herpes simplex virus.

**Figure 4.**
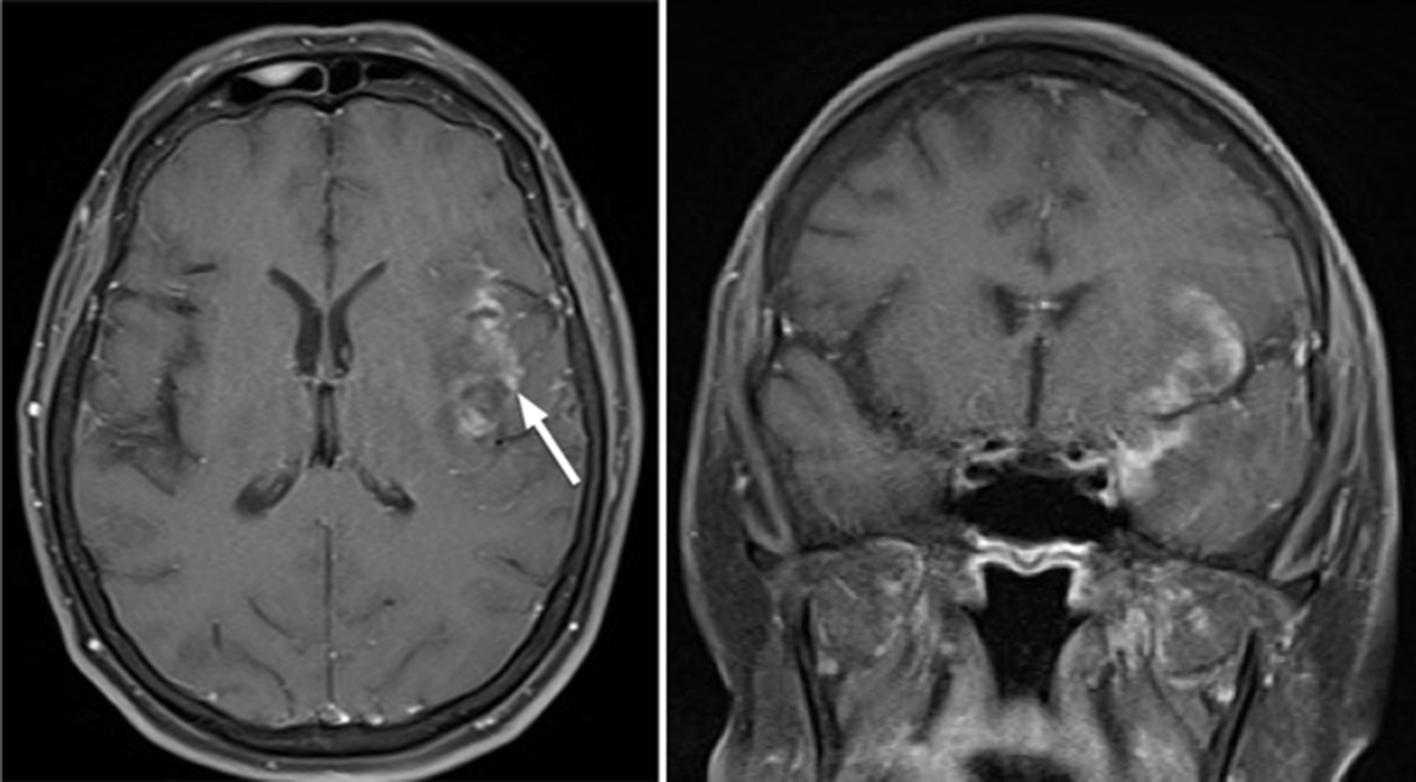
Axial (left) and coronal (right) T1 weighted post contrast sequences show left insular signal abnormality. There is heterogeneous parenchymal enhancement after contrast administration but no discrete masses or nodules.

An MRI performed in the acute phase, on day three of admission, identified asymmetrical cortical and subcortical signal hyperintensity involving the left insula, hippocampus, temporal stem and anterior temporal pole on T2 weighted MR sequences (Figure 2). Subtle ipsilateral mesial temporal lobe and subfrontal hyperintensity were also identified. Confluent insular haemorrhage was visible on susceptibility-weighted sequences with parenchymal enhancement in areas of signal abnormality (Figures 3 and 4). The basal ganglia and deep white matter were spared.

Six days after admission the patient had a generalised tonic-clonic seizure. Progressive petechial haemorrhage was visible on repeat CT corresponding to the area of parenchymal abnormality. Progressive neurological symptoms were documented with new mild right-sided neglect and 4/5 right upper limb weakness. The patient was prescribed a loading dose of phenytoin and commenced on levetiracetam with good effect.

## Management

In total, 3 weeks of i.v. acyclovir were administered. Several days after cessation of acyclovir, low-grade fevers returned. No new source of infection was found on further investigation. White cell count and C-reactive protein remained static, chest X-ray, urinalysis, stool culture and clinical signs remained unchanged. Fevers spontaneously resolved after 14 days.

Intensive speech and language therapy and physiotherapy was initiated with a multifaceted allied health professional rehabilitation approach. Slow, steady improvement in severity of dysphasia and limb weakness was observed over the subsequent 6 weeks. The patient was discharged after 2 months with continued outpatient rehabilitation, clobazam and levetiracetam for seizure-control and regular outpatient follow-up.

## Further Imaging

Nine months after initial diagnosis the patient reported progressive, left frontotemporal headaches and worsening of expressive dysphasia in parameters where progress had previously been documented. Analgesia had little effect on persistent headaches, which had slowly worsened over the preceding 4 weeks. The patient remained afebrile and seizure frequency remained static.

**Figure 5.**
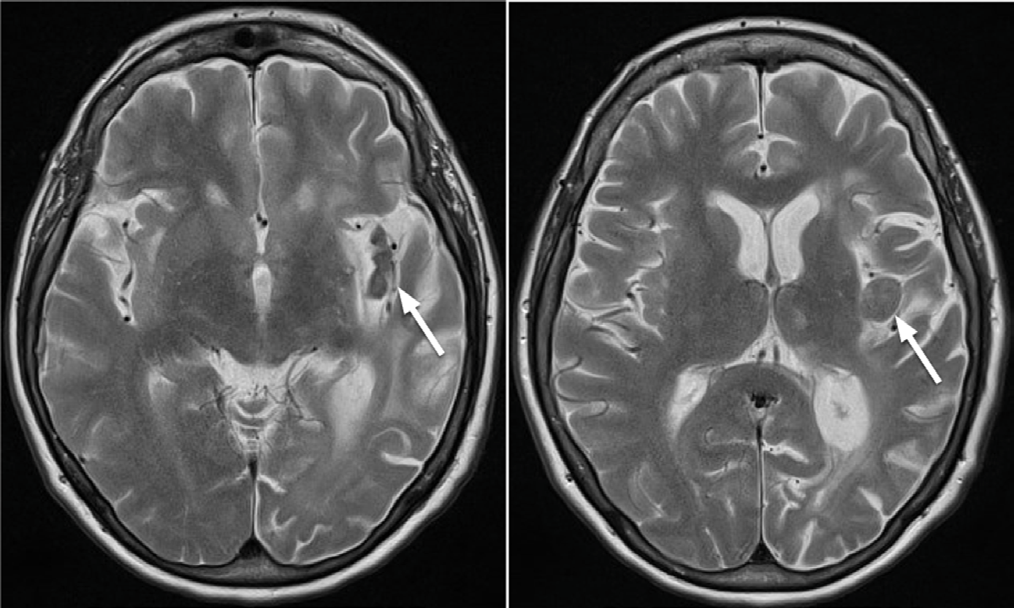
Axial T2 sequences from repeat MRI imaging performed 9 months after the acute presentation identified T2 hypointense coalescing nodules measuring up to 17 mm in the left insular region on a background of insular gliosis with parenchymal volume loss.

**Figure 6.**
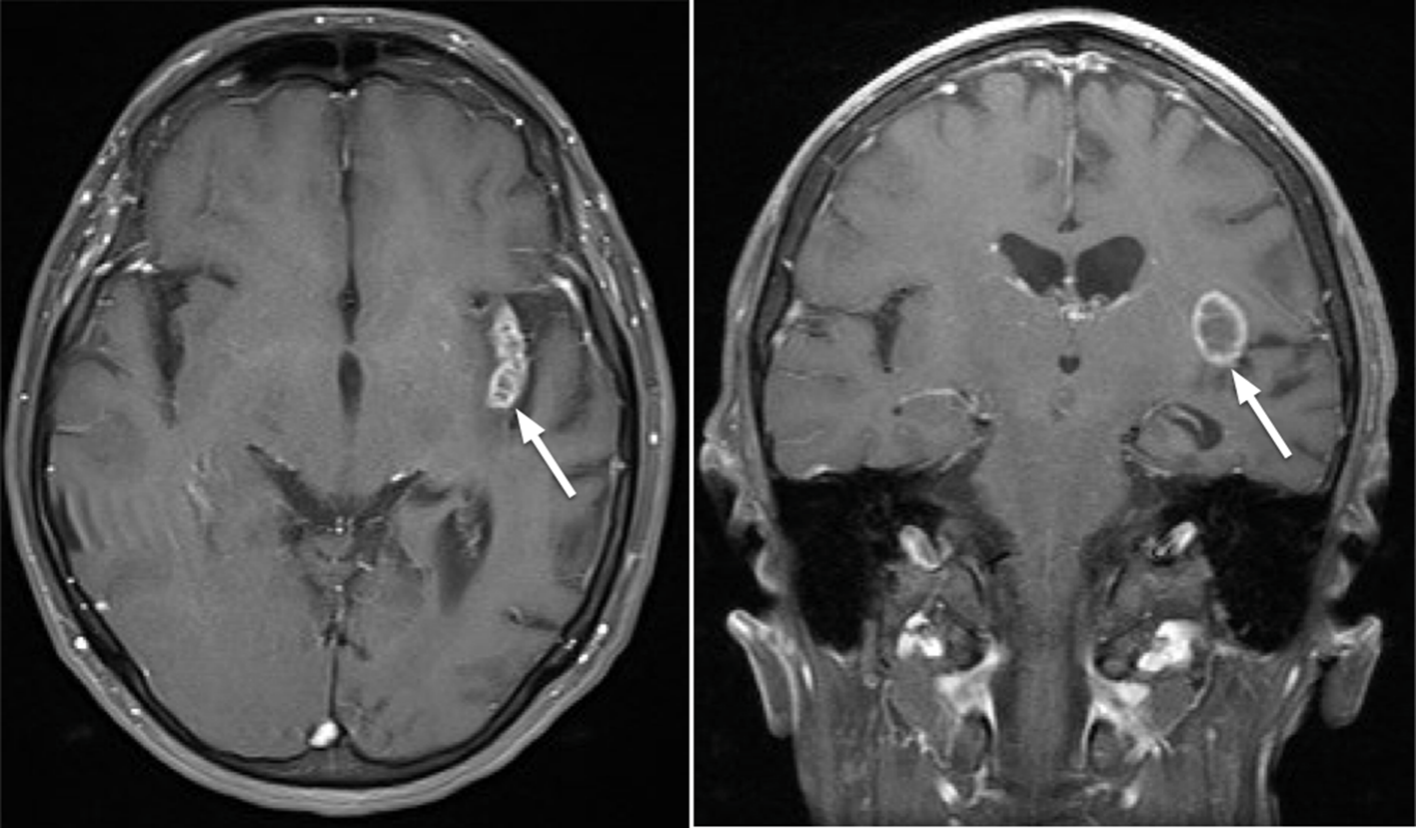
Axial (left) and coronal (right) contrast enhanced T1 weighted sequences performed 9 months after first acute presentation. Linearly arranged coalescing nodules in the insular region show mild peripheral and internal enhancement.

Repeat MRI brain imaging was performed. Centred on the atrophic left insula were linearly arranged heterogeneously enhancing “mass-like” mildly T2 hypointense nodules measuring up to 17 mm (Figures 5 and 6). The insular cortex and adjacent mesial temporal structures were atrophic with ex-vacuo dilatation of the temporal horn of the lateral ventricle. A lumbar puncture was negative for HSV-1 DNA on repeat viral PCR testing of CSF and CSF protein was within normal range measuring 0.44 g l^−1^. I.v. acyclovir was recommenced for 14 days. Within 7 days headaches had almost entirely resolved and a modest improvement in dysphasia was documented by the inpatient speech rehabilitation team, patient and family. The patient was discharged home and continued to make steady progress with rehabilitation.

## Outcome

Long-term outpatient clinical follow-up is ongoing. Headache symptoms have entirely resolved and right upper limb weakness remains mild (1/5) with minimal ipsilateral pronator drift. Moderate expressive and receptive dysphasia persist but have improved since readmission. The patient uses a computer to aid with communication. Seizures are well controlled on levetiracetam.

**Figure 7.**
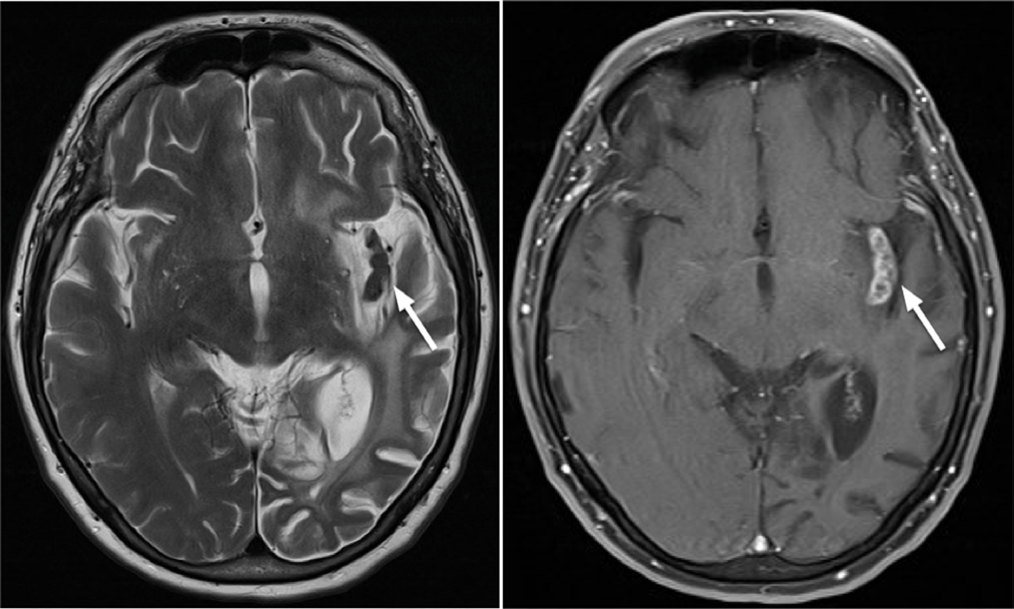
Axial T2 (left) and axial T1_ _weighted post-contrast sequences (right) performed 18 months after initial presentation identified progressive insula and temporal lobe encephalomalacia and volume loss with persistence of enhancing left insular granulomas which remain stable in size and morphology.

**Figure 8.**
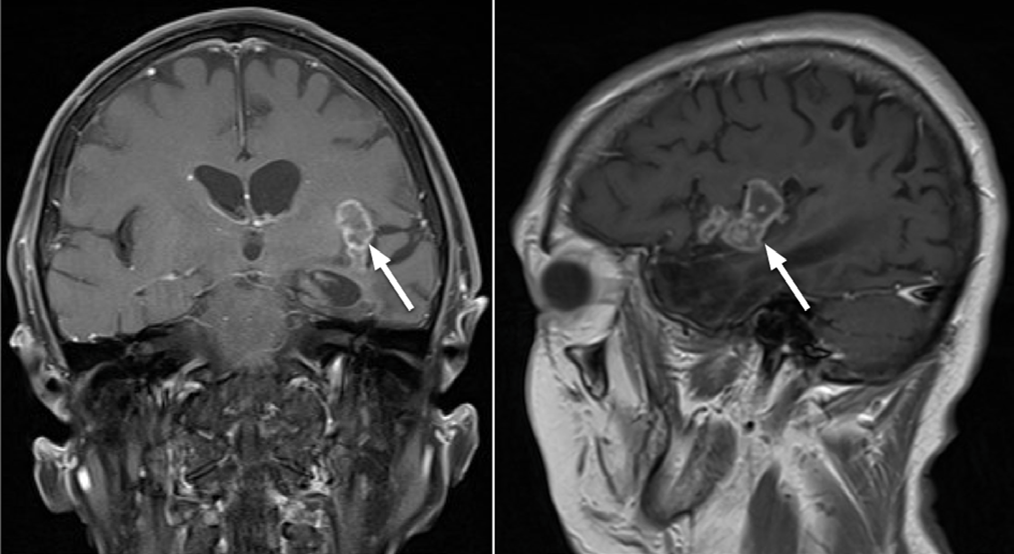
Coronal T2 (left) and Sagittal T1 weighted post-contrast sequence (right) performed 2 years after initial acute presentation of HSV encephalitis showed ongoing persistence of left insular granulomas, which remain stable in size and morphology. HSV, herpes simplex virus.

Contrast enhanced MRI brain, for monitoring purposes, was performed at 18 months (Figure 7) and 2 years after the initial diagnosis (Figure 8). Imaging findings remain entirely stable with no change in appearance or progression of nodular left insular enhancement.

## Discussion

A small proportion of patients with herpes simplex encephalitis develop a protracted or chronic course of disease. Clinical deterioration occurs after an initial period of improvement, with recurrent symptoms or signs identified weeks or months after the initial illness; an ostensible biphasic pattern of disease. Occasionally, relapse manifests many years after the initial diagnosis. Intractable seizures and progressive neurological deficits are frequently described in cases of chronic recurrent and chronic granulomatous encephalitis. Fever and altered levels of consciousness are also often documented.^6, 7^ This biphasic pattern of disease has been previously reported and is usually seen with recurrence in older children after early childhood, or neonatally acquired HSV encephalitis. Schutz et al noted that all published cases of biphasic chronic granulomatous HSV encephalitis were documented to have occurred in children.^8^ Our literature review is concordant with the assertion that chronic granulomatous encephalitis is nearly always described in children.^4–6,8,9^ Rare cases of histologically confirmed chronic granulomatous herpes encephalitis occurring in adults have been reported.^10^ The present case of granulomatous HSV encephalitis occurred in a 61-year-old female with no prior history of childhood HSV infection. The initial infection preceded the subsequent MR documentation of nodular granulomatous inflammation by only 9 months, raising the possibility of a monophonic progressive disease course, rather than a true biphasic illness. This apparently monophasic course was also reported by Varatharaj in adult granulomatous herpes encephalitis.^10^

Similar to previous clinical reports of chronic granulomatous HSV encephalitis our patient re-presented with persisting headaches and seizures. Imaging identified focal enhancing parenchymal lesions, to the author’s knowledge, a finding not previously demonstrated in adults.^9,11–13^ CSF PCR was negative for HSV DNA, a common finding in the context of chronic HSV granuloma formation. CSF viral DNA load is frequently very low or absent in chronic granuloma formation related to HSV.^8^

In this case, enhancing granulomas were identified on a background of insular gliosis and parenchymal calcification. The combination of an enhancing mass, signal abnormality and surrounding oedema should raise concern for a neoplastic process, with biopsy and histological assessment suggested in ambiguous cases, particularly where CSF PCR is negative. Biopsy also offers the opportunity to confirm the presence of granulation tissue in cases where focal nodules are not identified. The patient described in our case was a Jehovah’s witness and reluctant to undergo an invasive procedure. The degree of diagnostic certainty for granuloma formation was considered high in our case given the recent, confirmed diagnosis of HSV encephalitis and the persisting co-location of the new abnormality within the area of prior involvement. Histological confirmation with biopsy was not pursued in this case, therefore the diagnosis, although strongly suspected, was not histologically confirmed. The patient remains symptomatically stable and lesions remain entirely unchanged on long-term serial imaging over subsequent years.

Previous reports describing late relapse concluded that reactivation of latent virus was the likely cause of recurrence.^14^ The propensity to develop chronic granulomatous HSV encephalitis has been linked to underlying immunocompromise; in potential clinical scenarios which include corticosteroid treatment, immunodeficiency syndromes and treatment with chemotherapy or radiotherapy.^15^ No such background of immunocompromise was identified in our case. In addition, host differences may contribute towards the propensity to develop chronic granulomatous inflammation including polymorphism of the Toll-like receptor 3 pathway (TLR-3).^9^ Toll-like receptors form part of the innate immune system being activated by exogenous microbial pathogens and critical for antiviral immunity.^16^ TLR-3 is expressed in the central nervous system and is thought essential for natural immunity to HSV-1. Zhang et al postulated that neurotropic viruses such as HSV-1 had contributed to maintenance of the TLR-3 allele in evolution.^17^ Zhang et al described a genetic propensity for patients with TLR-3 deficiency to develop chronic HSV encephalitis. Guo et al described single-gene inborn errors of TLR-3 dependent activation of interferon mediated immunity.^18^ The Toll-like receptor 3 status was not tested in our patient. However, knowledge of the TLR-3 status may be helpful in future cases to identify patients requiring long-term treatment. Although the administration of acyclovir is currently considered the only modifiable prognostic factor,^19^ the value of corticosteroid therapy is also under investigation with an ongoing prospective clinical trial.^20^

## Conclusion

Herpes simplex encephalitis may rarely induce a chronic granulomatous reaction with headache and intractable seizures the most common presenting clinical features. Enhancing “mass-like” granulomatous nodules are rarely identified on MR brain imaging. CSF PCR is frequently negative for the detection of viral DNA in this context. Chronic granulomatous encephalitis is almost exclusively reported in children. We report a very rare case of suspected adult chronic nodular granulomatous encephalitis.

## Learning points

1. Herpes simplex encephalitis is the most common sporadic viral encephalitis in the Western world and remains one of the most devastating infections of the central nervous system. The disease typically follows an acute monophonic course. Uncommonly, patients with herpes simplex encephalitis relapse with recurrent symptoms or signs weeks, months or years after the initial infection.
2. Chronic granulomatous herpes encephalitis is almost exclusively described in children, with rare accounts describing the disease process in adults. Rarer still, is the formation of nodular “mass-like” granulomas in the affected area of parenchyma.
3. Analysis of cerebrospinal fluid in cases of chronic granulomatous herpes encephalitis is commonly negative for detection of herpes simplex viral DNA when utilising polymerase chain reaction detection techniques.
4. Host differences may contribute towards the propensity to develop chronic granulomatous inflammation such as polymorphism of the Toll-like receptor 3 pathway. Toll-like receptors form part of the innate immune system. TLR-3 is expressed in the central nervous system and is thought to be essential for natural immunity to HSV-1.
